# Seroepidemiology and Molecular Characterization of Hepatitis E Virus in Macaca Mulatta from a Village in Yunnan, China, where Infection with this Virus Is Endemic 

**DOI:** 10.5812/kowsar.1735143X.730

**Published:** 2011-09-01

**Authors:** Fen Huang, Wenhai Yu, Xiuguo Hua, Shenrong Jing, Weikun Zeng, Zhanlong He

**Affiliations:** 1 Faculty of Life Science and Technology, Kunming University of Science and Technology, Kunming, China; 2 Institute of Medical Biology, Chinese Academy of Medical Sciences and Peking Union Medical College, Kunming, China; 3 Zoonosis Laboratory, School of Agriculture and Biology, Shanghai Jiao Tong University, Shanghai, China

**Keywords:** Hepatitis E virus, Macaca Mulatta, Swine, Seroepidemiologic, Molecular Characterization

## Abstract

**Background:**

Hepatitis E virus (HEV) infection is a significant public health concern and has been identified as a zoonotic infection.

**Objectives:**

Since no reports have characterized the epidemiological and genotypic features of HEV infections in Macaca mulatta (rhesus macaques) from Yunnan, China, where swine HEV infections are endemic, we aimed to investigate these characteristics.

**Materials and Methods:**

Seroepidemiological and molecular characterization of HEV in both Macaca mulatta and pigs from the Yunnan province of China were conducted using enzyme-linked immunosorbent assay (ELISA) and reverse transcription-nested PCR (RT-nPCR). Four hundred and eighty-two stool samples (320 from Macaca mulatta and 162 from pigs) and 92 serum samples (all from Macaca mulatta) were collected for the detection of HEV RNA and anti-HEV antibodies (IgG/IgM).

**Results:**

Thirty-three rhesus macaques (35.87%) were positive for HEV IgG. Of these, 3 were also positive for HEV IgM. Four different strains of swine HEV RNA were detected in pigs; however, we failed to detect any in Macaca mulatta.

**Conclusions:**

Results indicate that Macaca mulatta may not be a natural reservoir of HEV.

## 1. Background

Hepatitis E virus (HEV) infection is a significant global public health concern and is associated with particularly high mortality rates in pregnant women [1]. HEV is transmitted primarily by the fecal-oral route or through contaminated water [2][3]. It can also be transmitted across species between humans, pigs, boars, deer, chickens, and rabbits [3][4][5]. HEV antibodies have been found in pigs, rats, cats, and cattle [6][7][8][9], with pigs identified as the most important reservoirs. Evidence has shown that veterinarians working with pigs were at increased risk of acquiring HEV infection [10]. In 2010, swine HEV was isolated from a village in the rural city of Kunming, where nonhuman primates are housed. Therefore, investigation of the epidemiology of HEV in Macaca mulatta in this region is necessary. Macaca mulatta is a commonly used animal model in the evaluation of the efficacy of HEV vaccines, the pathogenesis of HEV infections, and other studies to investigate HEV, such as xenotransplantation [11][12]. Organ transplant recipients have been reported to be at risk of HEV infection, and thus, the study of xenotransplantation in Macaca mulatta may lead to the development of beneficial therapeutics to avoid HEV infection during organ transplantation [12]. Although the Yunnan province has the most diverse population of wild animals in China, epidemiological and genotypic data for HEV are lacking.

## 2. Objectives

We sought to immediately investigate the epidemiology of HEV in Macaca mulatta following the detection of genotype 4 swine HEV RNA in the rhesus macaques in a village in Yunnan.

## 3. Materials and Methods

### 3.1. Stool and SerumSamples

Fresh stool samples (162 from pigs and 320 from Macaca mulatta) and serum samples (from 92 rhesus macaques) were separately collected between 2008 and 2011. The samples were stored at -70°C until use.

### 3.2. Detection of HEV RNA

Stool specimens were suspended at 10% w/v in phosphate-buffered saline (PBS; pH 7.4), containing 0.01% di-ethyl pyrocarbonate (DEPC), and centrifuged at 12000 × g for 10 min. Total RNA was extracted from the supernatant of each stool sample and serum sample with TRIzol® reagent (Invitrogen, USA) according to the manufacturer’s instructions. Reverse transcription was performed using a reverse transcriptase kit (AMV XL for RT-PCR; Takara, Japan) according to the manufacturer’s directions. Previously described HEV-specific primers were used [10]; these included the forward primer (P1) 5′-AAT TAT GCY CAG TAY CGR GTT G-3′ and the reverse primer (P2) 5′-CCC TTR TCY TGC TGM GCA TTC TC-3′, and internal primers, which included the forward primer (P3) 5′-GTW ATG CTY TGC ATW CAT GGC T-3′and the reverse primer (P4) 5′-AGC CGA CGA AAT CAA TTC TGT C-3′. These primers had been previously confirmed to detect all 4 known mammalian HEV genotypes. The expected RT-nPCR product was 348 bp. The RT-PCR protocol was carried out by incubation at 42°C for 30 min, followed by 85°C for 5 min. The resulting cDNA was amplified by nested PCR at 94°C for 2 min, followed by 39 cycles of 94°C for 1 min, 42°C for 1 min, and 72°C for 1 min, with a final incubation at 72°C for 7 min. The PCR products were detected both by electrophoresis on agarose gel containing 0.5 μg/mL ethidium bromide and by sequencing on a DNA analyzer (Applied Biosystems 3730 DNA Analyzer; Invitrogen, USA).

### 3.3. Detection of Anti-HEV IgG and IgM Antibodies

Serum samples were tested for the presence of HEV-specific IgG and IgM by using commercial ELISA kits (Wantai, China) containing recombinant ORF2 peptides from the HEV genome as well as both positive and negative controls. The sensitivity and specificity of the kits have been previously reported [13][14]. Sera were tested in duplicate according to the manufacturer’s directions, with cutoff values for IgG and IgM assays set at 0.22 and 0.26, respectively and also determined based on the mean OD450 values from the negative controls (± standard deviation).

### 3.4. Sequence and Phylogenetic Analysis

The nucleotide sequences of the amplified PCR products and of prototypes of different genotypes of HEV strains were aligned using MEGA 3.0 software (version 3.0, http:// www.megasoftware.net). The genomic sequences of prototype HEV strains were obtained from GenBank. Phylogenetic trees were generated by the minimum evolution and interior branch methods. Bootstrapping with 1,000 resamplings of the data was performed to calculate branch percentages. The identity between nucleotide sequences was calculated using the MegAlign program (DNAstar package version 5.03; Lasergene, DNAstar Inc., Madison, WI, USA).

### 3.5. Statistical Analysis

Statistical analysis was performed using SPSS statistical software. The descriptive statistics have been reported. A P value of < 0.05 was considered statistically significant.

## 4. Results

### 4.1. Detection of Anti-HEV IgG and IgM Antibodies in Sera of Macaca Mulatta

Thirty-three (35.87%) serum samples from Macaca mulatta were positive for anti-HEV IgG antibodies, while only 0.44% was positive for HEV IgM (3/92). Fifty percent (21/42) of females were positive for anti-HEV IgG antibodies, but only 24% (12/50) of males tested positive (Table 1). The rate of anti-HEV IgG antibody detection was significantly higher in females than in males (P > 0.01), which suggested that females may be more sensitive to HEV infection than males. No significant differences were found in the rate of infection in different age groups, with 41.25% of adolescents testing positive (age, 1 to 4 years), 31.25% of adults testing positive (age, 5 to 20 years), and 40% of elders testing positive (age, greater than 20 years, Table 1.

**Table 1:** Serological Investigation of HEV in Macaca Mulatta

	1–4, y (Adolescent)	5–19,y (Adult)	≥ 20, y (Elder)	Male	Female
Number	39	48	5	50	42
HEV-positive	16	15	2	12	21
Positive rate (%)	41.3	31.25	40	24	50

### 4.2. Detection of HEV RNA

HEV RNA was detected by RT-nPCR. The HEV positive rate was 2.5% (4/162) in swine stool samples; however, we failed to detect HEV RNA both in stool and serum samples for Macaca mulatta.

**Figure 1:**
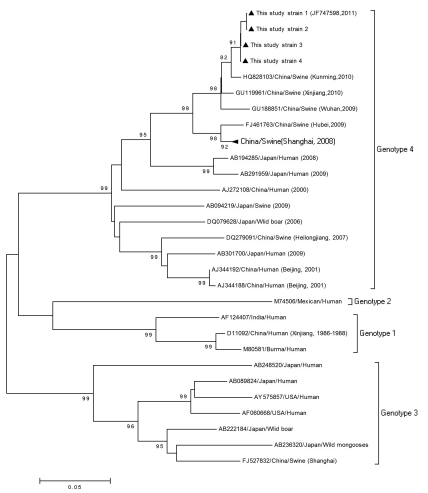
Phylogenetic Analysis Based on the ORF2 (350 bp) Sequence of the Isolates in This Study and 25 Other References of 4 HEV Genotypes, Using the Neighbor- Joining Method

### 4.3. Phylogenetic Analysis

The 4 swine HEV strains isolated from the village were highly homologous (98.3–99.4%), and shared high similarity (98%) with the strain isolated from a slaughterhouse in the city of Kunming last year (GenBank: HQ828103, Figure 1 ). These swine strains share 97% similarity with the strain isolated from Xinjiang province in 2009 (GenBank: GU119961), and 96% similarity with the Wuhan isolate (GenBank: GU188851), also from 2009. Phylogenetic analysis suggested that swine HEV strains prevalent in China have remained highly conserved in recent years. When compared with the reference HEV isolates,

homology was above 79% for genotype 1 (GenBank: M80581), 80% for genotype 2 (GenBank: M74506), 77% for genotype 3 (GenBank: AY575857), and 94% for genotype 4(GenBank: EU034709). One of the sequences in this study was submitted to GenBank (JF747598).Phylogenetic analysis clearly illustrated that all HEV sequences could be divided into 4 distinct genotypes, as was found in previous studies [14][15]. The HEV sequences isolated in the present study clearly belonged to genotype 4 and formed a cluster together with the Kunming, Xinjiang, and Wuhan strains Figure 1.

## 5. Discussion

Pigs serve as an important reservoir for HEV, and exposure to pigs may pose a risk of zoonotic infection. Wild rhesus macaques captured from the rural region of the Yunnan province quite often come in contact with both pigs and humans. Although Macaca mulatta has been frequently inoculated (either orally or intravenously) with human, swine or avian HEV strains to serve as animal models in HEV studies, whether it generally carries anti-HEV antibodies or HEV RNA has remained unknown. The present study provided data to describe the seroepidemiology of HEV in Macaca mulatta and the molecular characterization of HEV in pigs in the Yunnan province of China from samples collected between 2008 and 2011. Although the percentage of rhesus macaques testing positive for anti-HEV IgG antibodies (35.87%) was lower than that of wild rats (44–90%, [7]) or pigs (66.4–81.6%, [16][17]), it was higher than that of humans (21.1%, [14][16]), cattle (18.7%), and sheep (12.4%) [16]]. The prevalence of anti-HEV IgG antibody suggested that Macaca mulatta may be frequently exposed to an HEV-like antigen, as is observed in dogs, rats, and cats [8][18]. Few rhesus macaques (0.44%, 3/92) were positive for HEV IgM, which indicated that the HEV-like antigen was still prevalent in this area. However, we failed to detect HEV RNA in both serum and stool samples from these macaques by a universal RT-PCR assay that is capable of detecting genetically divergent strains of HEV.

Swine HEV strains prevalent in this area were highly conserved throughout most of the swine HEV strains in China. However, only 2.5% of pigs tested in this study were positive for HEV RNA, a value much lower than that seen in other provinces in China, such as Shanghai (7%, 39/554, [19]) and Beijing (22.89%, 19/83, [20]). Phylogenetic analysis revealed that the HEV isolate from this study clustered with the Chinese swine HEV strains but were distinct from Japanese strains of human HEV and most strains of swine HEV from other countries. The most prevalent viruses are the genetically diverse genotype 4 viruses, and the HEV strains isolated from different geographic regions of the world are genetically heterogenic. The monkeys sampled in this study were originally captured from the wild, suggesting that HEV infection might have been acquired from contact with HEV-infected wild boars, wild rats, or humans.In conclusion, the current study suggests that anti-HEV IgG antibodies are widely prevalent in Macaca mulatta populations in the Yunnan province of China.Phylogenetic analysis has shown that this genotype 4 HEV isolate clustered with Xinjiang and Wuhan strains, which are common HEV strains prevalent in China.
